# Visible-Light-Driven Z-Type Pg-C_3_N_4_/Nitrogen Doped Biochar/BiVO_4_ Photo-Catalysts for the Degradation of Norfloxacin

**DOI:** 10.3390/ma17071634

**Published:** 2024-04-03

**Authors:** Yi Li, Wenyu Wang, Lei Chen, Huifang Ma, Xi Lu, Hongfang Ma, Zhibao Liu

**Affiliations:** 1 School of Environmental Science and Engineering, Qilu University of Technology (Shandong Academy of Sciences), Jinan 250353, China; 2 School of Chemistry and Chemical Engineering, Qilu University of Technology (Shandong Academy of Sciences), Jinan 250353, China

**Keywords:** Z-scheme heterojunction, PCN/N-Biochar/BiVO_4_, photocatalysis, norfloxacin

## Abstract

Antibiotics cannot be effectively removed by traditional wastewater treatment processes, and have become widespread pollutants in various environments. In this study, a Z-type heterojunction photo-catalyst Pg-C_3_N_4_ (PCN)/Nitrogen doped biochar (N-Biochar)/BiVO_4_ (NCBN) for the degradation of norfloxacin (NOR) was prepared by the hydrothermal method. The specific surface area of the NCBN (42.88 m^2^/g) was further improved compared to BiVO_4_ (4.528 m^2^/g). The photo-catalytic performance of the catalyst was investigated, and the N-Biochar acted as a charge transfer channel to promote carrier separation and form Z-type heterojunctions. Moreover, the NCBN exhibited excellent performance (92.5%) in removing NOR, which maintained 70% degradation after four cycles. The main active substance of the NCBN was •O_2_^−^, and the possible degradation pathways are provided. This work will provide a theoretical basis for the construction of heterojunction photo-catalysts.

## 1. Introduction

The extensive use and production of antibiotics has led to widespread antibiotic pollution in various environments [1]. The removal of antibiotics in conventional wastewater treatment processes is difficult [2], which can pose a potential hazard to the aquatic environment [3].

Various treatment techniques have been investigated and applied to remove or degrade antibiotics from wastewater, such as adsorption [4,5], biological treatment processes [6], electrochemical advanced oxidation [7], photo-catalytic oxidation [8], and Fenton oxidation [9]. Among them, photo-catalytic technology has become one of the most popular new technologies for various applications, due to its convenience and environmental friendliness. In the presence of light, the electrons in the catalyst valence band absorb light energy and transition to the conduction band, leaving strongly oxidizing holes in the valence band [10]. Previous studies have reported a variety of low-cost catalysts, including metal oxides, sulfides, carbides, halides, and hydroxides [11,12]. Among them, the semiconducting metal oxides, especially bismuth-based metal oxides, such as bismuth vanadate (BiVO_4_), are considered to be promising materials as they respond in visible light. The strong charge transfer of bismuth-based metal oxides can cause superior photo-catalytic efficiency in visible light [13,14].

Bismuth vanadate (BiVO_4_) has attracted great interest due to its excellent properties, low band gap, good dispersion, non-toxicity, corrosion resistance, and outstanding photo-catalytic effect in the degradation of organic pollutants under visible light irradiation [15]. It is an N-type semiconductor with good chemical and light stability. In addition, the advantages of flexible optical and electronic properties (band gap of about 2.4 eV) make it an attractive choice for solar energy harvesting [16]. BiVO_4_ has three crystal structures, including a tetragonal zircon phase (t-z), a monoclinic scheelite phase (m-s), and a tetragonal scheelite phase (t-s). Moreover, the m-s structure exhibits better photo-catalytic performance in visible light, due to the lone-pair distortion of the Bi 6s orbitals in the BiVO_4_ semiconductor. Meanwhile, the overlap of O 2p and Bi 6s orbitals in the valence band (VB) is favorable for the migration of photo-generated charge carriers, thus improving the photo-catalytic activity [17]. However, the narrow bandgap energy of BiVO_4_ can cause the rapid complexation of photo-induced carriers. To solve this problem, the method of carbon surface modification has been proved to be an effective strategy to improve photo-catalytic performance [18].

Most carbon-based materials come from non-renewable fossil fuel sources. By comparison, biochar produced by slow pyrolysis of waste biomass is inexpensive, readily available, and renewable, making it a high-quality source of carbon-rich materials [19]. Moreover, N-Biochar can provide lone-pair electrons of pyridine-N and pyrrole-N to participate in the redox reaction, which can improve the catalytic activity of biochar and has been applied in oxygen reduction catalysis [20]. On the one hand, the sufficient -OH and -NH_2_ functional groups of N-Biochar can effectively adsorb various pollutants. On the other hand, biochar can be used as a platform for loading various catalytic nanoparticles. In recent years, metal oxide and biochar composites have received much attention for pollutant adsorption and photo-catalysis [21]. Various biochar-loaded photo-catalysts have been reported with better sensitivity to visible light, higher stability, regeneration ability, and photo-catalytic degradation efficiencies [22,23]. Guo et al. [24] prepared a composite of biochar with BiVO_4_, which could improve the degradation efficiency of sulfanilamide. Wei et al. [25] prepared a composite of N-Biochar with BiVO_4_, which demonstrated better efficiency in removal of triclosan.

A new semiconductor material could be introduced to form a heterojunction, to further improve the performance of the above-mentioned metal oxide and biochar composite photo-catalytic materials (such as N-Biochar and BiVO_4_). Kang et al. [26] designed an enhanced Z-type heterojunction using bismuth tungstate/bismuth iodide loaded on biochar to remove tetracycline, which promoted the directional separation of photo-generated carriers and realized the efficient utilization of photogenic charge. Cheng et al. [27] designed biochar/2ZIS/WO_3_ photo-catalytic materials with heterojunction to improve the charge separation efficiency and photo-catalytic performance of the catalysts. In addition, graphitic carbon nitride (g-C_3_N_4_) has been widely studied as a promising photo-catalyst due to its chemical stability and large specific surface area [28,29]. The conjugated graphite structure within the carbon-based skeleton can interact with the g-C_3_N_4_ to form a stable composite material [30], which can overcome the weak interaction (van der Waals forces) between adjacent C-N layers in g-C_3_N_4_, resulting in a high degree of complexation of photo-generated carriers. Thus, a novel Z-type ternary semiconductor-conductor-semiconductor heterojunction can be constructed using N-Biochar, BiVO_4_, and g-C_3_N_4_ with better photo-catalytic performance.

Norfloxacin (NOR) is a broad-spectrum antimicrobial agent of the fluoroquinolone class, which can form complexes with metal ions such as Ca^2+^, Mg^2+^, Fe^3+^, and Al^3+^ to acquire long-term stability in the environment. In addition, it is one of the most frequently detected antibiotics in sediments [31]. Thus, NOR was investigated as the target pollutant in this study.

The aim of this study was to synthesize a novel ternary composite catalyst and analyze its performance for NOR degradation. Moreover, the stability of the synthesized catalyst and the contribution of active species was studied. In addition, the intermediate products of NOR and the degradation pathway were clarified to discuss the removal mechanism of the Z-type catalyst. Thus, this study will provide a reference for the construction of heterojunction photo-catalysts.

## 2. Experiment

### 2.1. Materials and Reagents

The reeds for producing biochar were acquired from Jinan, China, and dried at 60 °C. The ammonium metavanadate (NH_4_VO_3_, AR, 99%), nitric acid (HNO_3_, AR, 65%), ammonia solution (NH_3_•H_2_O, AR, 25%), urea (CH_4_N_2_O, AR, 99%), thiourea (CH_4_N_2_S, AR, 99%), sodium hydrogen carbonate (NaHCO_3_, AR, 99.5%), methanol (CH_4_O, AR, 99.5%), isopropanol (C_3_H_8_O, AR, 99.7%) and trichloromethane (CHCl_3_, AR, 99%) were purchased from Sinopharm. The hydrochloric acid (HCl, AR, 37%) was purchased from Yantai Yuandong Fine Chemicals Co., Ltd. (Yantai, China). The bismuth nitrate pentahydrate (Bi(NO)_3_•5H_2_O, AR, 99%) was purchased from Macklin. All reagents are used directly in this study.

### 2.2. Synthesis of Photo-Catalysts

#### 2.2.1. Preparation of Nitrogen Doped Biochar (N-Biochar)

N-Biochar was prepared according to a previous study [32]. The reed powder was passed through a 60 mesh sieve, then mixed with urea and NaHCO_3_ in a mass ratio of 1:4:3, and ground evenly in a mortar. Then, the mixture was heated to 700 °C at an elevated temperature rate of 5 °C/min^−1^ under N_2_ atmosphere and stored for 1 h. Later, the black powder was soaked with HNO_3_ at ambient temperature for 1 h. The mixture was then filtered and the solids were washed with deionized water to remove soluble salts until the pH reached 7. Then, they were dried overnight at 80 °C to obtain N-Biochar.

#### 2.2.2. Preparation of N-Biochar/BiVO_4_ (NCB)

The NCB was prepared by a hydrothermal method [24]. Bi(NO_3_)_3_•5H_2_O and NH_4_VO_3_ with a molar ratio of 1:1 were dissolved in 2 M HNO_3_ and NH_3_•H_2_O, respectively. Then, the NH_4_VO_3_ solution was slowly poured into the Bi(NO_3_)_3_•5H_2_O solution under magnetic stirring until a yellow precipitate was produced. The pH of the mixture was adjusted to 7 with NH_3_•H_2_O and stirred for 0.5 h (Suspension A). Next, different mass fractions (10%, 20%, and 30%) of N-Biochar were added to the yellow solution and sonicated for 0.5 h, and the suspension was magnetically stirred for 1 h. The suspension was sealed and heated at 180 °C for 12 h. After natural cooling, it was washed with ultrapure water and then dried at 70 °C for 6 h. Then, the products were labeled as NCB-10%, NCB-20%, and NCB-30%. In addition, the BiVO_4_ was also synthesized in parallel with the above method except without N-Biochar addition. NCB-20% was selected for further study due to having the best degradation effect among the three fractions (Figure S1).

#### 2.2.3. Preparation of Pg-C_3_N_4_/N-Biochar/BiVO_4_ (NCBN)

The Pg-C_3_N_4_ (PCN) was prepared by mixed pyrolysis of urea and thiourea [33]. A mixture of 10 g of urea and 3 g of thiourea powder was placed in a muffle furnace and heated at 550 °C for 2 h. Different mass fractions (3%, 5%, and 10%) of PCN and N-Biochar were added together into Suspension A. Then, the suspension was ultrasonicated for 0.5 h and stirred for 1 h, and heated at 180 °C for 12 h in a stainless-steel reactor. After natural cooling, it was washed with ultrapure water and dried at 70 °C for 6 h. The products were numbered NCBN-3%, NCBN-5%, and NCBN-10%. The specific synthesis steps was shown in Figure S2.

### 2.3. Characterization

The methods used to characterize the catalysts are described in the Supporting Material (Text S1).

### 2.4. Photo-Catalytic Tests

The NOR was degraded in a photochemical reactor under visible light irradiation, and the photo-catalytic performance of NCBN was also evaluated. The degradation efficiency of NCBN on NOR at different dosages can be seen in Figure S3. Catalyst (25 mg) was added to the 50 mL NOR solution (10 mg/L) under dark conditions for 0.5 h to reach the adsorption-desorption equilibrium. Then, the reaction system was transferred to simulated daylight radiation with a 300 W Xe lamp, and 3 mL of the solution was taken at regular intervals. Moreover, the solution was passed through a 0.42 µm filter membrane to analyze the removal efficiency of NOR by UV-Spectrophotometer (278 nm). In addition, the intermediates of NOR degradation were analyzed by a Shimadzu LCMS-9030 system. The mobile phases were formic acid and acetonitrile (0.1% formic acid) at a flow rate of 0.3 mL/min, and a C18 column (2.1 × 100 mm, 3 µm particle size) was used.

### 2.5. Free Radical Trapping Experiments

The NCBN composites were used for free radical trapping experiments to determine the effect of different actives on photo-catalytic performance. Isopropyl alcohol (IPA), methanol (MT), and trichloromethane (CHCl_3_) were used as scavengers of hydroxyl (•OH), hole (H^+^), and superoxide radicals (•O_2_^−^).

## 3. Results and Discussion

### 3.1. Physicochemical Properties of the Photo-Catalysts

**Figure 1:**
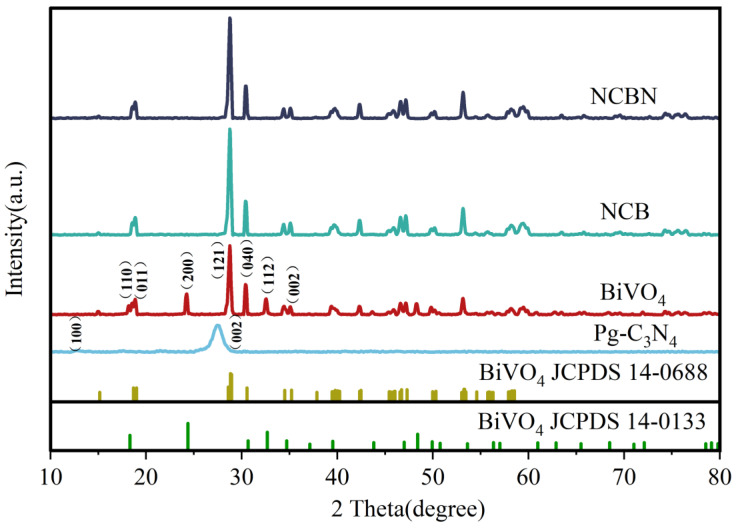
XRD patterns of the BiVO_4_, Pg-C_3_N_4_ (PCN), Nitrogen doped biochar (N-Biochar)/BiVO_4_ (NCB), and PCN/N-Biochar/BiVO_4_ (NCBN).

The crystal structure of the synthesized catalyst was analyzed by XRD (Figure 1). The characteristic peaks of BiVO_4_ were indexed to a mixed structure of monoclinic scheelite (JCPDS No. 14-0688) and tetragonal zircon (JCPDS No. 14-0133), indicating that the BiVO_4_ had been successfully prepared. The strong peak at 28.75° was consistent with the (121) crystal plane of monoclinic BiVO_4_ [34], which also suggested that monoclinic scheelite was the main structure of BiVO_4_. Meanwhile, the characteristic peaks of NCB and NCBN showed a monoclinic scheelite structure, which might be due to the coupling interaction between BiVO_4_ and biochar. For PCN, the small peak near 12.7° was consistent with the (100) crystal face, which was caused by the plane stacking of triple homogeneous triazine units. Also, the strong peak at 27.4° corresponded to the (002) crystal face of PCN, which originated from an interlayer aromatic structure [35]. In both NCB and NCBN, due to the disruption of the PCN structure, the intensity of the PCN peaks decreased significantly, indicating that the development of the composite photo-catalyst was different from that of the original PCN in the plane surface [36].

**Figure 2:**
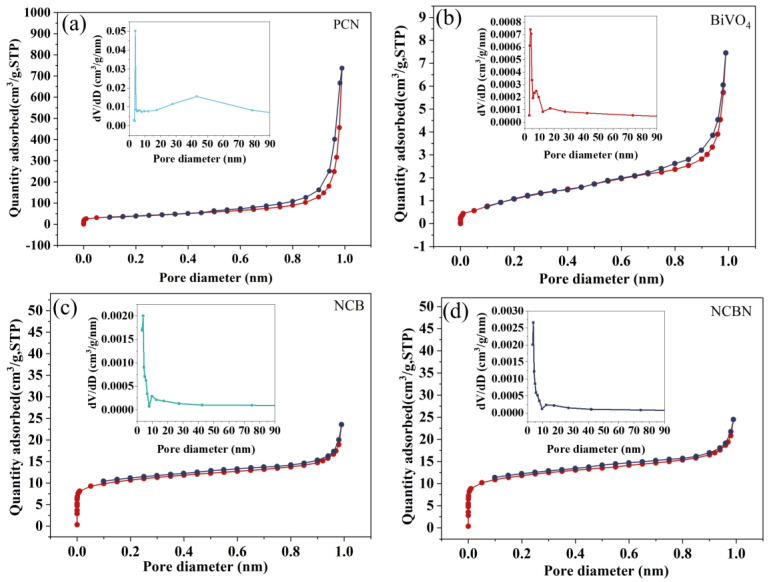
N_2_ adsorption–desorption isotherms and pore size distributions of (**a**) PCN, (**b**) BiVO_4_, (**c**) NCB, and (**d**) NCBN.

The specific surface area and porosity of photo-catalysts affects their photo-catalytic performance [37]. The N_2_ adsorption–desorption curves and pore size distributions of PCN, BiVO_4_, NCB, and NCBN are shown in Figure 2. According to the classification of the International Union of Pure and Applied Chemistry (IUPAC), all four samples belong to type IV isotherms as well as H3 hysteresis loops. According to the pore size distribution map, most of the pore sizes were between 2–50 nm, especially between 2–10 nm, which were mostly of a mesoporous structure (2–50 nm). Table 1 shows details of the specific surface area, pore size, and pore volume for all samples. Compared with pure BiVO_4_ (4.528 m^2^/g) and NCB (38.85 m^2^/g), the specific surface area of the NCBN (42.88 m^2^/g) was increased. The reason was that a small amount of C_3_N_4_ was added, indicating that its capacity for adsorption of the pollutant was stronger. Although the specific surface area of PCN (138.9 m^2^/g) was relatively large, the photo-catalytic performance of NCBN was superior to that of PCN due to its efficient charge transfer.

**Figure 3:**
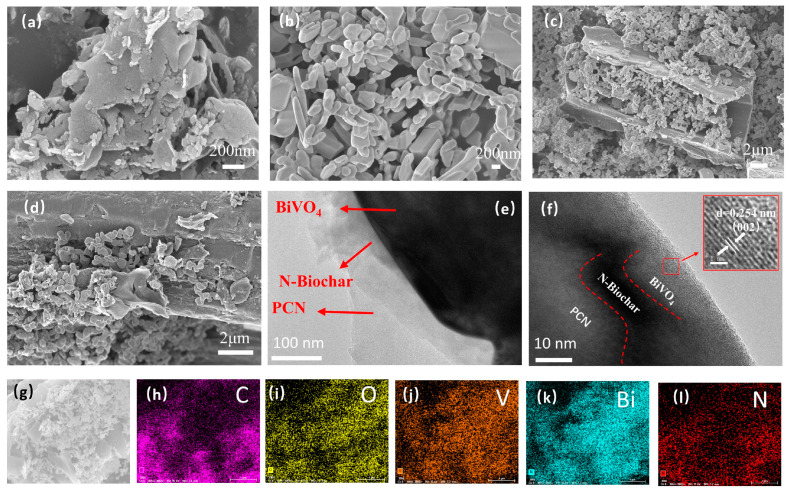
SEM images of (**a**) PCN, (**b**) BiVO_4_, (**c**) NCB, and (**d**) NCBN; TEM images of (**e**) NCBN, (**f**) HR–TEM of NCBN, and (**g**–**l**) SEM–EDS elemental mapping images of NCBN.

The morphology of the synthesized catalysts was examined by SEM. As shown in Figure 3a, the prepared PCN with cracks and pores between the layers had a thin and loose layered structure, and the interlayer nanostructures were superimposed on each other. Moreover, Figure 3b indicates that the BiVO_4_ exhibited a monoclinic scheelite and tetragonal zircon phase structure in NCB. In addition, the BiVO_4_ mainly presented a monoclinic scheelite structure and was uniformly distributed on the surface of the biochar (Figure 3c). In the preparation of the NCBN, because the stacked PCN nanosheets were peeled off after stirring, sonication, and hydrothermal treatment, the BiVO_4_ was bound to the biochar (Figure 3d).

TEM and HR-TEM were used to study the different compositions of the material. As shown in Figure 3e, the PCN nanosheets were almost transparent, and BiVO_4_ and N-Biochar were bound to the PCN. In Figure 3f, the lattice stripe width of 0.254 nm corresponds to the (002) crystal plane of the monoclinic scheelite structure BiVO_4_. The dark amorphous part corresponds to N-Biochar, and the PCN exhibited weak crystallinity due to its amorphous structure. The EDS spectrum (Figure 3g–l) and Figure S4 show the presence of elements C, N, Bi, O, and V in the NCBN, and the five elements were uniformly distributed in the NCBN.

**Figure 4:**
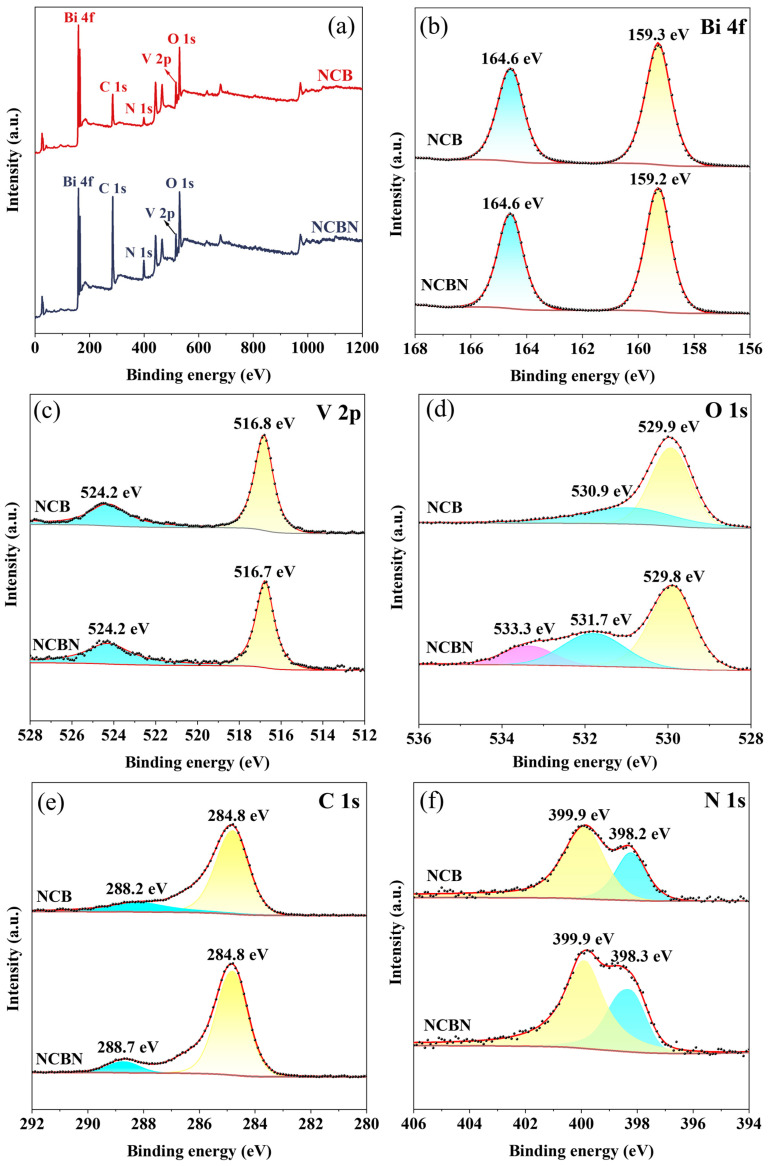
XPS spectra of NCB and NCBN: (**a**) full-survey spectrum, (**b**) Bi 4f, (**c**) V 2p, (**d**) O 1s, (**e**) C 1s, and (**f**) N 1s.

XPS was used to analyze and compare the elemental states and chemical composition of NCB and NCBN. As shown in Figure 4a, the prepared NCBN consisted of five elements (C, N, Bi, O, and V), which was consistent with the EDS spectra. Figure 4b–f shows the high-resolution spectra of the individual elements. As shown in Figure 4b, the Bi 4f of the NCB showed two strong peaks at 159.3 eV and 164.6 eV, corresponding to Bi 4f_7/2_ and Bi 4f_5/2_ for Bi^3+^ [38], respectively. However, the peak at 159.3 eV was negatively shifted to 159.2 eV in the NCBN. For V 2p (Figure 4c), the two peaks at 516.8 eV and 524.2 eV in NCB corresponded to V 2p_1/2_ and V 2p_3/2_, respectively, which could be attributed to V^5+^ spin-orbit splitting [39]. In the V 2p profile in NCBN, the peak at 516.8 eV was negatively shifted to 516.7 eV. For the O 1s spectrum (Figure 4d), the peak at 529.9 eV in the NCB was attributed to the oxygen atom in the V-O bond. In the NCBN material, this peak moved to 529.8 eV. Another O 1s peak at 531.76 eV and 533.3 eV in the NCB might originate from chemisorbed oxygen species and hydroxyl groups, respectively [40]. For the C 1s in NCB (Figure 4e), the two peaks at 284.8 eV and 288.2 eV were attributed to the C=C bond and -C=O [41]. In the NCBN spectrum, 288.2 eV was positively shifted to 288.7 eV. In Figure 4f, the NCB shows two peaks at 398.2 eV and 399.9 eV, which were attributed to pyridine nitrogen and pyrrole nitrogen, respectively [42]. Similarly, the peak at 398.2 eV was positively shifted to 399.3 eV in the NCBN.

Compared with NCB, the peaks of Bi 4f, O 1s, and V 2P of NCBN were shifted toward negative binding energies, and C 1s and N 1s were shifted toward positive binding energies. In general, a positive shift of the peak indicated a decrease in the surface electron density, and a negative shift indicated an increase in the surface electron density. This demonstrated that compared with NCB, there was a decrease in electrons on the N-Biochar surface and an increase in electrons on the BiVO_4_ surface in NCBN. This differs from the previous mechanism for NCB, suggesting that the strong interactions between BiVO_4_, PCN, and N-Biochar in NCBN lead to changes in the chemical environment [25]. Moreover, the increase of the number of electrons in BiVO_4_ and the decrease of the number of electrons in N-Biochar might be due to the fact that N-Biochar acted as an electronic bridge, facilitating the complexation of electrons in PCN and holes in BiVO_4_. XPS analysis illustrated the successful synthesis of Z-type heterojunctions.

**Figure 5:**
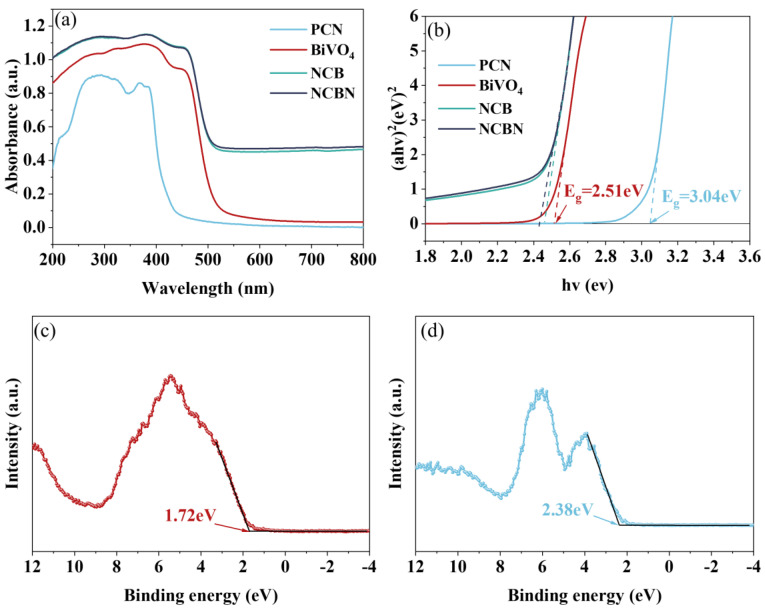
(**a**) UV–vis DRS, (**b**) the Tauc plots of the materials, and the VB–XPS spectra of (**c**) BiVO_4_ and (**d**) PCN.

The optical properties and band gap depletion (E_g_) of materials were characterized by UV DRS. As shown in Figure 5a, the absorption edge of PCN was about 420 nm, and the absorption limit of BiVO_4_ was about 510 nm. It was noteworthy that the absorbed light range of both NCB and NCBN was shifted towards the visible range compared to PCN and BiVO_4_. Moreover, the NCBN showed the widest absorbed light range and the strongest absorbance. The reason for this phenomenon was that the heterojunction altered the optical properties of the material. The E_g_ of all samples was calculated by the Tauc equation (Equation (1)). In Figure 5b, the E_g_ of BiVO_4_ and PCN are shown as 2.51 eV and 3.04 eV, respectively. However, the E_g_ of NCBN was relatively low, which was conducive to its visible light response. In addition, the VB-XPS spectra (Figure 5c,d) were used to determine the valence band (VB) positions of BiVO_4_ and PCN, which were 1.72 eV and 2.38 eV, respectively. According to Equation (2), the conduction band (CB) position of BiVO_4_ and PCN was −0.79 eV and −0.66 eV, respectively.

(1) $${\left(\alpha h\nu \right)}^{n}=A\left(h\nu -E_{g}\right)$$

(2) $$E_{CB}=E_{VB}-E_{g}$$

where A, h, ν, and α are the scaling factor, Planck’s constant, optical frequency, and absorption coefficient, respectively. For direct bandgap semiconductors n = 2, and for semiconductors with indirect bandgap halves n = 1/2 [43].

### 3.2. Photo-Catalytic Properties

#### 3.2.1. Photo-Catalytic Degradation of NOR

**Figure 6:**
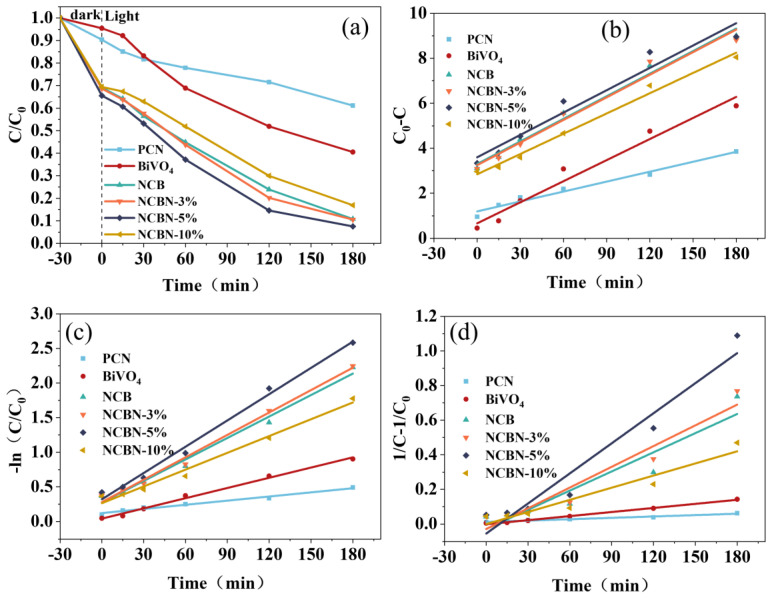
(**a**) Photocatalytic degradation efficiency of NOR, and (**b**–**d**) 0, 1, 2-order kinetic curves for the NOR degradation.

The fabricated samples were degraded with NOR under visible light, and the photo-catalytic properties of the materials were evaluated by the degradation of NOR. As shown in Figure 6a, after stirring in a dark environment for 30 min, the adsorption of NCBN on NOR reached 35.5%, and adsorption-desorption equilibrium was achieved. Moreover, its adsorption performance was much higher than that of pure BiVO_4_ and PCN. This was because the introduction of biochar increased the specific surface area and adsorption sites. Compared with NCB, the slightly improved adsorption effect of NCBN might be due to the introduction of PCN. In addition, a 300 W Xe lamp was fitted with a 420 nm cutoff filter and used to irradiate the samples for 3 h. The degradation rates of BiVO_4_, PCN, NCB, and NCBN were 59.5%, 38.9%, 89%, and 92.5%, respectively. Among them, NCBN exhibited the highest photo-catalytic performance, the degradation efficiency of NCBN gradually increased with the increase of PCN content, and the degradation efficiency of NCBN-5% was the highest.

The degradation rate of NOR was analyzed by the 0, 1, and 2 order kinetic equations. As shown in Figure 6b–d and Table 2, the R^2^ values of the 1-order correlation coefficients for the six samples (PCN, BiVO_4_, NCB, NCBN-3%, NCBN-5%, and NCBN-10%) were 0.9816, 0.9938, 0.9873, 0.9918, 0.9921, and 0.9846, respectively. These values were higher than those in the 0-order and 2-order kinetic equations, which indicated that the degradation of NOR conformed to the 1-order kinetic equation. In addition, the kinetic constants k for the six samples (PCN, BiVO_4_, NCB, NCBN-3%, NCBN-5%, and NCBN-10%) were 0.0020, 0.0050, 0.010, 0.011, 0.013, and 0.0080 min^−1^, respectively. The degradation rate constant of NCBN-5% was higher than that of other materials, which further proved that NCBN-5% had higher degradation efficiency.

In addition, the activity of the prepared photocatalysts in NOR degradation was compared with several published papers (Table 3). It can be seen that the NCBN had excellent photocatalytic performance compared with other photocatalysts.

#### 3.2.2. Stability and Recyclability of Photo-Catalytic Materials

**Figure 7:**
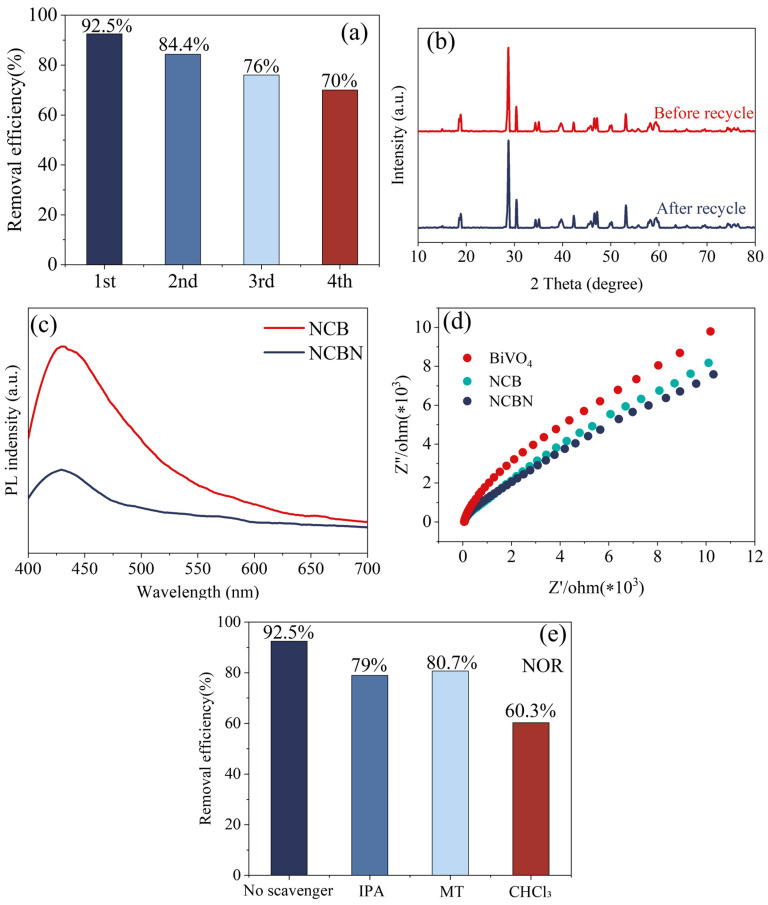
(**a**) Recycling times on the photocatalytic activity, (**b**) XRD pattern of the material before and after cycling, (**c**) PL spectra, (**d**) EIS Nyquist plots of the electrochemical impedance spectra curves, and (**e**) the quenching experiment of NCBN catalysts.

The stability and recyclability of photo-catalytic materials also plays a crucial role in practical applications. Figure 7a shows the stability of NCBN while degrading NOR over four cycles. Each cycle consisted of dark adsorption of 30 min and visible light exposure of 180 min, and the NCBN was cleaned and dried after the completion of each cycle. After four cycles, the removal of NOR remained above 70%, which indicated that the stability of NCBN was high. In addition, Figure 7b shows the XRD pattern of the material before and after cycling. There was no significant difference between the samples prepared before and after cycling, which indicated that the structure and the crystalline shape of NCBN did not change during use. These phenomena show the good recyclability of NCBN.

### 3.3. Photo-Catalytic Mechanism

#### 3.3.1. Photoluminescence, Electrochemical Properties

The migration and separation efficiency of photogenerated electrons and holes in the material affects the performance of photo-catalysts. These properties were investigated by photoluminescence (PL) spectrum and electrochemical impedance spectroscopy (EIS). In Figure 7c, the PL peak at about 430 nm was attributed to the complexation of photogenerated electrons and holes in the NCB and NCBN. Ou et al. [49] showed that the lower peaks represent higher separation efficiency and better photo-catalytic performance. Moreover, the peak of NCBN was significantly lower than that of NCB, indicating that the fluorescence intensity was weak and the recombination of electron and hole was inhibited in NCBN. In addition, the charge transfer resistance was studied by EIS (Figure 7d). Wang et al. [50] showed that the smaller semicircle indicated lower charge transfer resistance at the electrode interface, and better photoexcited electron-hole pair separation. The NCBN exhibited the smallest radius, which indicates more efficient charge transfer. These results showed that the heterojunctions of NCBN expanded the light absorption range and improved the charge transfer efficiency.

#### 3.3.2. Free Radical Bursts

To further understand the photo-catalytic mechanism, a quenching experiment of the active substance was carried out, and then the main active substances promoting NCBN degradation of NOR were determined. Moreover, IPA, MT, and CHCl_3_ were used as trapping agents for •OH, H^+^, and •O_2_^−^, respectively. As shown in Figure 7e, the degradation efficiency of NOR after the addition of scavengers was 79% (IPA), 80.7% (MT), and 60.3% (CHCl_3_), respectively. This result indicated that -O_2_^−^ played an important role in the degradation of NOR, followed by the active substances of •OH and H^+^.

#### 3.3.3. Degradation Intermediates and Pathways

**Figure 8:**
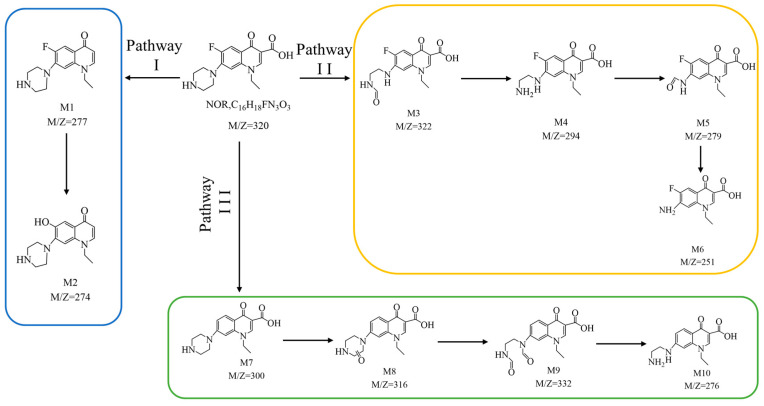
The possible degradation pathways of NOR.

The intermediates formed during the photo-catalytic degradation of NOR by NCBN were identified by LC-MS analysis. The liquid mass mapping and structural formulae of the intermediates are shown in Figure S5 and Table S1. The possible degradation pathways of NOR are presented in Figure 8. The basic structure of NOR is composed of the piperazine ring, quinolone group and benzene ring. Therefore, based on the identified intermediates, three possible degradation pathways for NOR were proposed. Pathway (I): the NOR was oxidized to remove the carboxyl group and produce M1 (M/Z = 277), then it was replaced by F to form the intermediate M2 (M/Z = 274). Pathway (II): after piperazine ring cleavage and decarbonylation, the NOR was attacked by the active species to form the intermediates M3 (M/Z = 322) and M4 (M/Z = 294). Then, the methylene amide group (-CH_2_NH_2_) on M4 was oxidized to the aldehyde group (-CO) of M5 (M/Z = 279), resulting in C-N cracking to form M6 (251). This was the central step in the pathway of piperazine ring rupture. Pathway (III): the NOR was defluorinated, and then the •OH attacked the carbon atom to form two intermediates, M7 (M/Z = 300) and M8 (M/Z = 316). The piperazine ring of M8 was opened, and the M9 (M/Z = 332) and M10 (M/Z = 276) were generated by dehydrogenation and deoxygenation reactions. Eventually, most of the M2, M6, and M10 were mineralized to CO_2_ and H_2_O.

#### 3.3.4. Photo-Catalytic Degradation Mechanism of NOR

**Figure 9:**
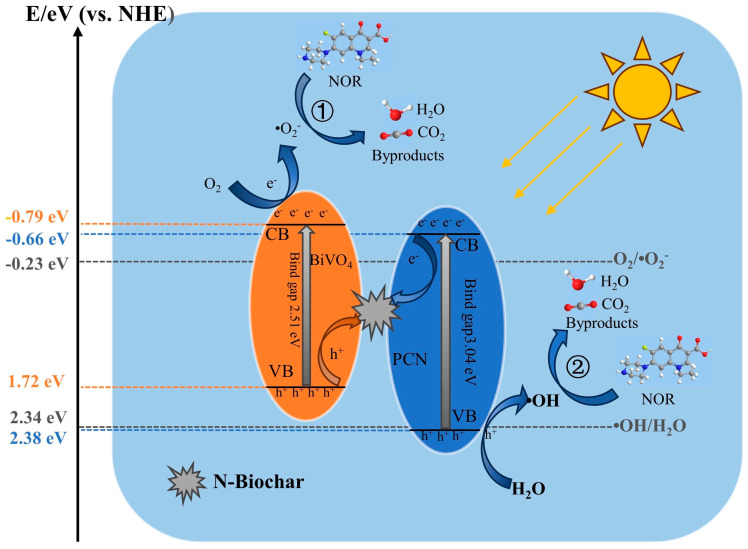
Schematic diagram of photocatalytic degradation mechanism.

The mechanism of NCBN degradation of NOR was proposed. As shown in Figure 9, a carrier complex center was established between BiVO_4_ and PCN due to the presence of N-Biochar. For N-Biochar, due to the similar size of nitrogen and carbon atoms, the nitrogen atoms replacing carbon atoms caused little damage to the skeleton structure of carbon materials, which could maintain the stability of carbon materials [51]. Meanwhile, the nitrogen atoms were more electronegative than carbon atoms, with the result that the doped carbon material had superior electronic conductivity [52]. This phenomenon promoted the flow of photogenerated electrons in the CB of PCN into the N-Biochar conductor. Then, to maintain the charge balance, the holes generated in the VB of BiVO_4_ rapidly migrated into N-Biochar, where they recombined with electrons. As a result, the holes were mainly clustered in the VB of PCN and the electrons were mainly concentrated in the CB of BiVO_4_, which facilitated the separation of charge carriers.

Compared with the redox potentials of O_2_/•O_2_^−^ (−0.33 eV) and •OH/H_2_O (2.34 eV), the more negative potential in the CB of BiVO_4_ (−0.79 eV) promoted the reduction of O_2_ to •O_2_^−^, and the more positive potential in the VB of PCN (2.38 eV) promoted the oxidation of H_2_O to •OH. Moreover, •O_2_^−^ and •OH were also used as active substances to participate in the degradation of reaction ① and reaction ②, respectively (Figure 9). In addition, according to the free radical quenching experiments, it could be found that •O_2_^−^ played an important role. These phenomena indicate that the construction of high-efficiency Z–type heterojunctions in NCBN could isolate the photogenerated carriers, and endow the carriers with strong redox ability, thereby improving the pollutant removal performance. Therefore, the key steps in the photo-catalytic reaction are summarized in Equations (3)–(11), and the main mechanism for degrading NOR was the free radical reaction of •O_2_^−^.

(3) $$BiVO_{4}+\mathrm{hv}\to e^{-}\left(BiVO_{4}\right)+h^{+}\left(BiVO_{4}\right)$$

(4) $$\mathrm{PCN}+\mathrm{hv}\to e^{-}\left(\mathrm{PCN}\right)+h^{+}\left(\mathrm{PCN}\right)$$

(5) $$h^{+}\left(BiVO_{4}\right)+\mathrm{NC}\to BiVO_{4}+h^{+}\left(\mathrm{NC}\right)$$

(6) $$e^{-}\left(\mathrm{PCN}\right)+\mathrm{NC}\to \mathrm{PCN}+e^{-}\left(\mathrm{NC}\right)$$

(7) $$h^{+}\left(\mathrm{NC}\right)+e^{-}\left(\mathrm{NC}\right)\to \mathrm{NC}$$

(8) $$e^{-}\left(BiVO_{4}\right)+O_{2}\to •O_{2}^{-}+BiVO_{4}$$

(9) $$•O_{2}^{-}\;+\;\mathrm{NOR}\to H_{2}O+CO_{2}\;+\;\mathrm{Byproducts}$$

(10) $$•\mathrm{OH}\;+\;\mathrm{NOR}\to H_{2}O+CO_{2}\;+\;\mathrm{Byproducts}$$

### 3.4. Economic Studies

In order to analyze the cost-effectiveness of the catalyst, the normalized efficiency of the catalyst was studied. The transformation number (TON) is usually defined as the quantity of pollutant in moles degraded by the per-unit mass of catalyst (mol pollutant/g_catalyst_); transformation frequency (TOF) is the quantity of pollutant in moles degraded by the per-unit time and unit mass of catalyst (mol pollutant/(g_catalyst_·min)); and the TOF divided by the input power of the light source (TOF/W) represents the cost-effectiveness [53]. As shown in Table 4, the cost of NCBN was similar to other catalysts, but the catalytic activity of NCBN was higher than other catalysts.

## 4. Conclusions

In this research, the NCBN ternary composites of PCN, BiVO_4_, and N-Biochar were successfully prepared by the hydrothermal method for the degradation of NOR. At a catalyst dosage of 500 mg/L and NOR dosage of 10 mg/L, the degradation rate of NOR was 92.5% after 3 h. The XRD, SEM, TEM, and XPS analyses showed that the NCBN was correctly prepared. Moreover, PL spectroscopy, EIS, and UV-vis DRS showed that NCBN had a wider range of absorbed light and more efficient charge transfer. According to the free radical quenching experiments, •O_2_^−^ played an important role in the degradation of NOR. In addition, the liquid chromatogram showed that during the photo-catalytic reaction, NOR was degraded and formed a series of new intermediates, most of which were eventually mineralized into CO_2_ and H_2_O. This research proves that it is feasible to improve the photo-catalytic performance of the material by introducing PCN to form a heterojunction, and the NCBN heterojunction catalyst will provide a reference for the construction of photo-catalysts.

## Figures and Tables

**Table 1:** The BET specific surface area, pore volume, and pore size of PCN, BiVO_4_, NCB, and NCBN.

Samples	BET Specific Surface Area (m^2^/g)	Pore Volume (cm^3^/g)	Pore Size (nm)
PCN	138.9	1.14	3.819
BiVO_4_	4.528	0.0110	3.822
NCB	38.85	0.0210	3.827
NCBN	42.88	0.0200	3.820

**Table 2:** Kinetic curve parameters for the NOR degradation.

Photo-Catalysts	0-Order		1-Order		2-Order	
	R^2^	k	R^2^	k/min^−1^	R^2^	k/(L·(mg·min)^−1^)
PCN	0.9769	0.015	0.9816	0.0020	0.9784	0.00020
BiVO_4_	0.9693	0.031	0.9938	0.0050	0.9956	0.00080
NCB	0.9861	0.034	0.9873	0.010	0.9035	0.0036
NCBN-3%	0.9742	0.034	0.9918	0.011	0.9383	0.0039
NCBN-5%	0.9547	0.033	0.9921	0.013	0.9453	0.0058
NCBN-10%	0.9903	0.0030	0.9846	0.0080	0.9333	0.0023

**Table 3:** Comparison of different photocatalysts for NOR degradation.

Catalyst	Catalyst Dose	NOR	Time	Degradation	Reference
LaOCl/LDH	0.4 g/L	10 mg/L	150 min	82.5%	[44]
BiOCl	1 g/L	10 mg/L	180 min	84%	[45]
M-BiVO_4_/T-BiVO_4_	1 g/L	20 mg/L	150 min	91%	[46]
ZnO/g-C_3_N_4_	1.43 g/L	8.61 mg/L	120 min	90%	[47]
BiVO_4_/GQDs/PCN	1 g/L	20 mg/L	120 min	86.3%	[48]
PCN/N-Biochar/BiVO_4_	0.5 g/L	10 mg/L	180 min	92.5%	This study

**Table 4:** Comparison of different catalysts for NOR degradation.

Catalyst	Catalyst Dose (g/L)	NOR Concentration (mg/L)	Light Source	% Degradation/Time (min)	TON (mol·g^−1^) × 10^−6^	TOF (mol·g^−1^·min^−1^) ×10^−8^	TOF/W (mol·g^−1^·min^−1^·W^−1^) × 10^−11^	Reference
LaOCl/LDH	0.4	10	Xenon (300 W)	82.5/150	3.2	2.1	7	[44]
BiOCl	1	10	Xenon (300 W)	84/180	1.3	0.72	2.4	[45]
BiVO_4_/GQDs/PCN	1	20	Xenon (300 W)	86.3/120	2.7	2.25	7.5	[48]
NCBN	0.5	10	Xenon (300 W)	92.5/180	2.89	1.6	5.3	This study

## Data Availability

Data are contained within the article.

## Supplementary Materials

The following supporting information can be downloaded at: https://www.mdpi.com/article/10.3390/ma17071634/s1, Figure S1: Effects of different ratios of N-Biochar on photocatalytic performance. Figure S2: Schematic diagram of the synthesis of the ternary PCN/N-Biochar/BiVO_4_ composite. Figure S3: Degradation efficiency of NCBN on NOR at different dosages. Figure S4: EDS spectrum. Figure S5: LC-MS analysis. Table S1: The information of organic intermediate product. Text S1: Characterization information.
