# Supplementary material for: Visible-Light-Driven Z-Type Pg-C3N4/Nitrogen Doped Biochar/BiVO4 Photo-Catalysts for the Degradation of Norfloxacin
Source: Materials (Basel). 2024 Apr 3;17(7):1634. doi: 10.3390/ma17071634 (PMC11012328; doi:10.3390/ma17071634)
Supplement: Supplementary file 1 [file materials-17-01634-s001.zip › materials-2935095-supplementary.pdf]

SUPPLEMENTARY INFORMATION

Visible-Light-Driven Z-Type Pg-C_3N_4/Nitrogen Doped Biochar/$BiVO_4$ Photo-Catalysts for the Degradation of Norfloxacin

Yi Li [1], Wenyu Wang [1,*], Lei Chen [1], Huifang Ma [1], Xi Lu [2], Hongfang Ma [1] and Zhibao Liu [2,*]

[1] School of Environmental Science and Engineering, Qilu University of Technology (Shandong Academy of Sciences), Jinan 250353, China
[2] School of Chemistry and Chemical Engineering, Qilu University of Technology (Shandong Academy of Sciences), Jinan 250353, China
* Correspondence: qluwwy@126.com (W.W.); emcsdjn@126.com (Z.L.)

This document consists of 8 pages, 1 text, 5 figures and 1 table.

Corresponding to section 2.2 in the pa-

Effects of different ratios of nitrogen-doped biochar onphotocatalytic performance

The effect of different ratios of N-Biochar on the NCB was evaluated by degrading

NOR as shown in Fig.S1. The NOR solution was configured by dissolving NOR (50 mg) into HCl (2.5 mL) to obtain 100 mg/L mother liquor. Then, 50 mL of the mother liquor was diluted into a 500 mL volumetric flask to obtain a 10 mg/L NOR solution. At this point, the initial pH of the solution is 3.5. Next, the catalyst (25 mg) was added to the NOR solution (50 mL) and stirred under dark conditions for 0.5 h, and the photocatalytic reaction was carried out under a 300 W xenon lamp with a 420 nm cutoff filter. As shown in Fig.S1(a), the catalytic rates of $BiVO_4$, NCB-10%, NCB-20%, and NCB-30% after 180 min of illumination were (K=0.005, 0.0066, 0.0103, 0.0032), respectively. The results show that the photocatalytic properties of the materials were further improved at higher percentages of nitrogen doped biochar (10 %-20 %). However, the photo-catalytic performance was limited at a higher percentage (30 %), because excessive N-Biochar led to a decrease in light penetration. Based on the above experiments, N-Biochar at a percentage of 20 % was determined as best suited for the synthesis of NCBN.

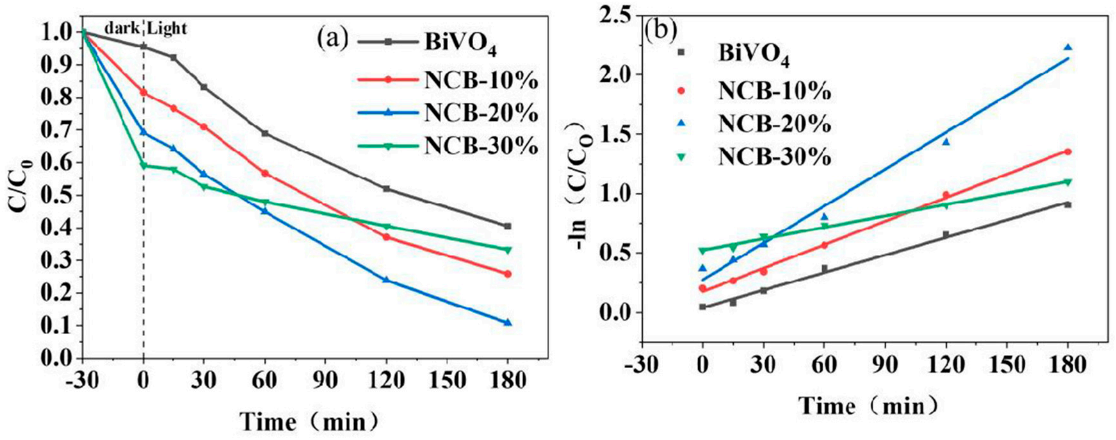

Figure S1: Effects of different ratios of N-Biochar on photocatalytic performance.

Corresponding to section 2.2.3 in the pa-

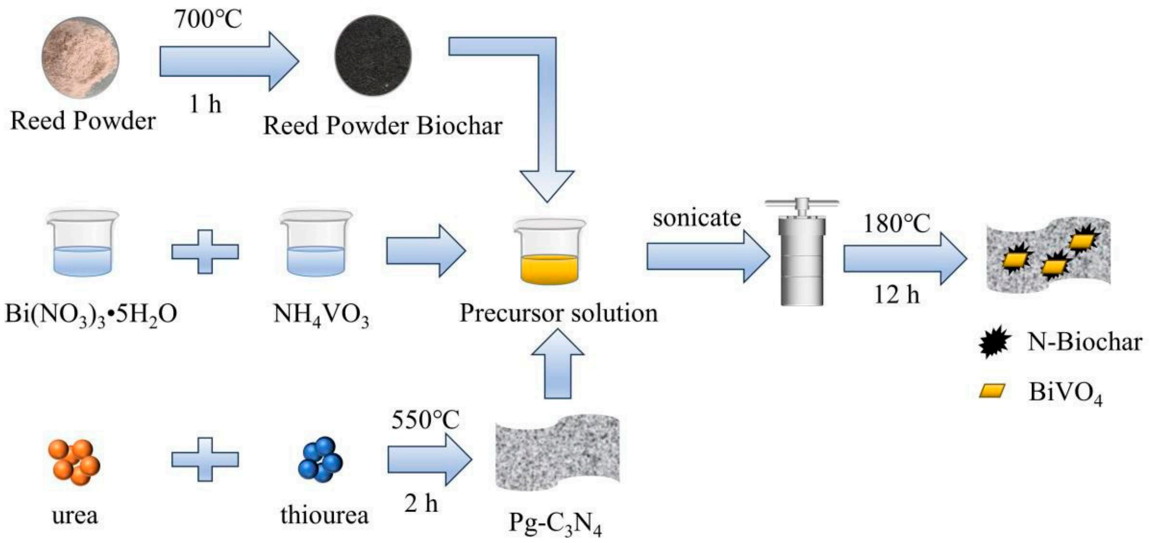

Figure S2. Schematic diagram of the synthesis of the ternary PCN/N-Biochar/BiVO4 composite.

Corresponding to section 2.4 in the pa-

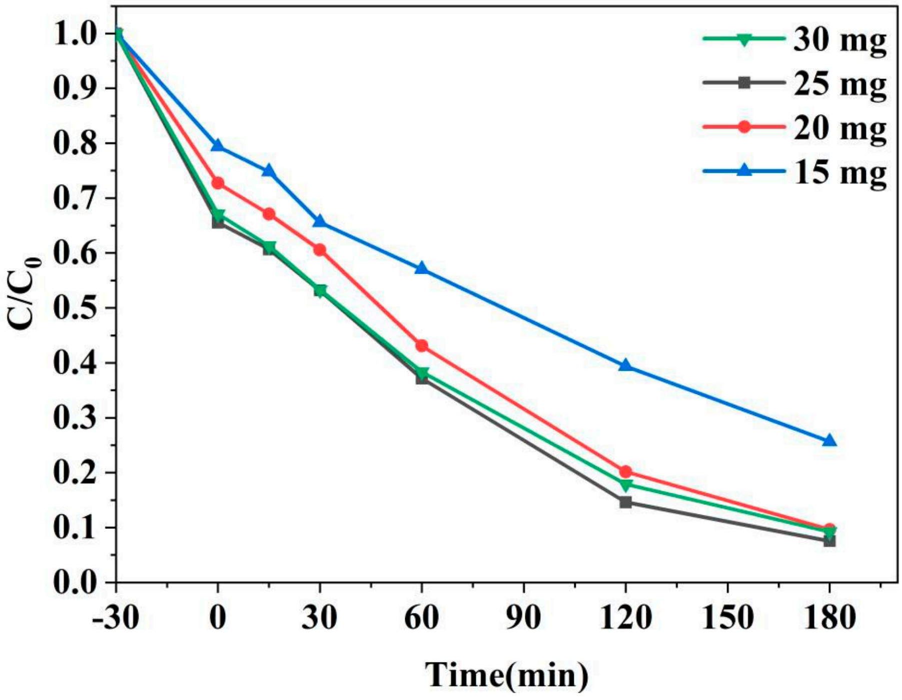

Figure S3. Degradation efficiency of NCBN on NOR at different dosages.

Corresponding to section 3.1 in the pa-

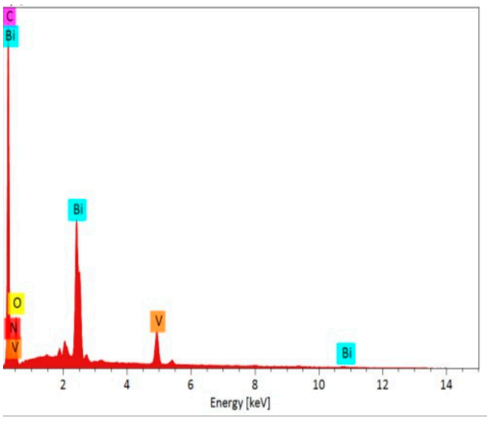

Figure S4. EDS spectrum.

Corresponding to section 2.3 in the pa-

Text S1

Characterization information

The crystal structure of the samples (2θ ranged from 5° to 80°) was analyzed by X-ray diffraction (XRD, Smartlab, Rigaku). The specific surface area and pore size distribution of the materials were analyzed by nitrogen adsorption-desorption isotherms (Autosorb-iQ-MP, Quantachrome Instruments, Boynton Beach, FL, USA). The microstructure of the prepared materials was obtained by scanning electron microscopy (SEM, ZeissG500). Energy dispersive X-ray spectroscopy (EDS) was used to detect the elemental distribution. The elemental analysis was performed using an X-ray photoelectron spectrometer (XPS, ESCALABXi+). The UV-visible diffuse reflectance (DRS) was obtained using a spectrophotometer (Shimadzu UV-3600i Plus). The photoluminescence spectra (PL) were tested on a steady-state transient fluorescence spectrometer (FLS1000). Then, the electrochemical impedance was tested on an electrochemical workstation.

Corresponding to section 3.3.3 in the pa-

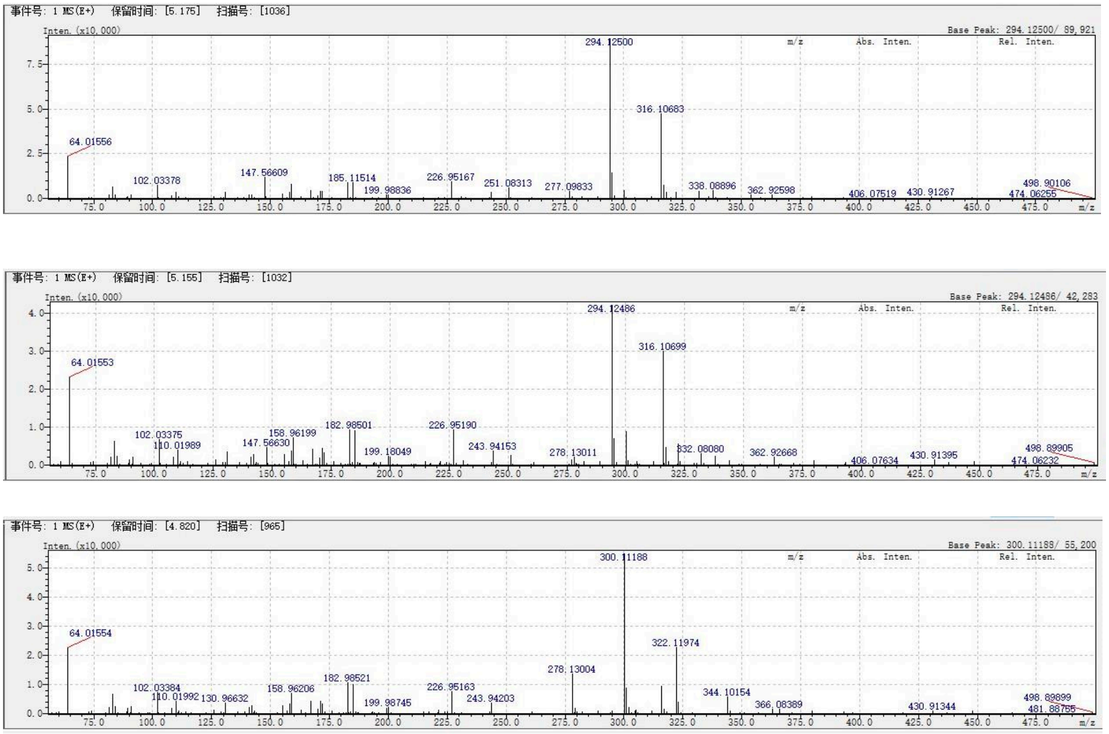

Figure S5: LC-MS analysis

Table S1. Details of organic intermediate products.

Products	M/Z	Probable structure
NOR	320	O
M1	277	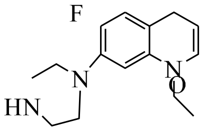
M2	274	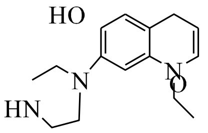
M3	322	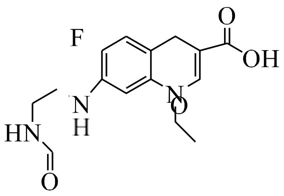

M4	294	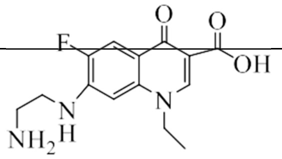
M5	279	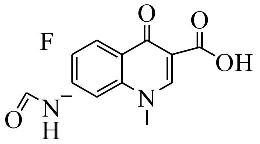
M6	251	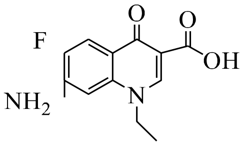
M7	300	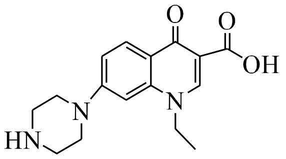
M8	316	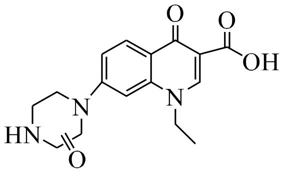
M9	332	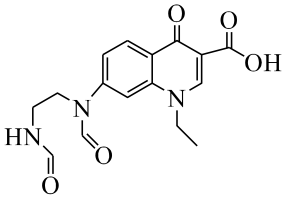
M10	276	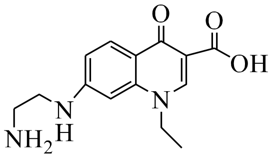
